# Disagreement in *F*_ST_ estimators: A case study from sex chromosomes

**DOI:** 10.1111/1755-0998.13210

**Published:** 2020-07-06

**Authors:** William J. Gammerdinger, Melissa A. Toups, Beatriz Vicoso

**Affiliations:** ^1^ Institute of Science and Technology Austria Klosterneuburg Austria

**Keywords:** *F*_ST_, sex chromosomes

## Abstract

Sewall Wright developed *F*_ST_ for describing population differentiation and it has since been extended to many novel applications, including the detection of homomorphic sex chromosomes. However, there has been confusion regarding the expected estimate of *F*_ST_ for a fixed difference between the X‐ and Y‐chromosome when comparing males and females. Here, we attempt to resolve this confusion by contrasting two common *F*_ST_ estimators and explain why they yield different estimates when applied to the case of sex chromosomes. We show that this difference is true for many allele frequencies, but the situation characterized by fixed differences between the X‐ and Y‐chromosome is among the most extreme. To avoid additional confusion, we recommend that all authors using *F*_ST_ clearly state which estimator of *F*_ST_ their work uses.

## 1 BACKGROUND

Genetic sex determination is common in both plants and animals (Bachtrog et al., 2014) and the pair of chromosomes where the sex‐determination gene resides are referred to as the sex chromosomes. Sex chromosomes are hypothesized to often emerge from autosomes once they have acquired a novel mutation for sex determination (Abbott, Nordén, & Hansson, 2017; Bachtrog et al., 2014). Linked, sexually antagonistic alleles can help to drive a novel sex‐determination allele to a higher frequency in the population (van Doorn & Kirkpatrick, 2010) and mechanisms that reduce recombination between sexually antagonistic loci and the novel sex‐determination locus are selectively favoured (Charlesworth, Charlesworth, & Marais, 2005; Rice, 1987). Due to the reduction in recombination, deleterious mutations accumulate and gradually decay the gene content within this region (Bachtrog, 2013; Blaser, Grossen, Neuenschwander, & Perrin, 2012). In some systems, large‐scale deletions or expansions of repetitive elements occur and lead to heteromorphic sex chromosomes (Bachtrog, 2013; Charlesworth et al., 2005). As a result of this process, sex chromosomes exist on a spectrum between harbouring a single nucleotide polymorphism (SNP) responsible for sex determination with no reduction in recombination from the surrounding region, as seen in fugu (Kamiya et al., 2012), to the highly decayed and heteromorphic sex chromosomes observed in many eutherian mammals (Bellott et al., 2014; Cortez et al., 2014).

Two common methods have been developed to identify sex chromosomes at different points on this spectrum using next‐generation sequencing data sets. In the more advanced stages of sex chromosome evolution the X‐ and Y‐chromosome share little genomic content. As a result, short reads from the Y‐chromosome align poorly to an X‐chromosome reference, resulting in a higher coverage in females than in males. These differences in coverage between males and females can be used to detect putatively nonrecombining regions of sex chromosomes (Fraïsse, Picard, & Vicoso, 2017; Huylmans, Toups, Macon, Gammerdinger, & Vicoso, 2019; Pal & Vicoso, 2015; Roesti, Moser, & Berner, 2013; Vicoso & Bachtrog, 2011, 2013, 2015; Vicoso, Kaiser, & Bachtrog, 2013). In the less advanced stages of sex chromosome evolution, the X‐ and Y‐chromosome differ only by a few base substitutions. Therefore, short reads from the X‐ and Y‐chromosome align in nearly equal proportions to an X‐chromosome reference and the identification of sex chromosomes instead relies on differences in allele frequencies in males and females (Böhne et al., 2019; Conte, Gammerdinger, Bartie, Penman, & Kocher, 2017; Dixon, Kitano, & Kirkpatrick, 2018; Fontaine et al., 2017; Gammerdinger, Conte, Acquah, Roberts, & Kocher, 2014; Gammerdinger, Conte, Baroiller, D’Cotta, & Kocher, 2016; Gammerdinger, Conte, Sandkam, Penman, & Kocher, 2019; Gammerdinger, Conte, Sandkam, & Ziegelbecker, 2018; Toups, Rodrigues, Perrin, & Kirkpatrick, 2018; Veltsos et al., 2019). Typically, SNPs are first identified among subpopulations of males and females, and regions with high levels of genetic differentiation between males and females are presumed to be sex‐linked. This genetic differentiation between males and females is often described in terms of *F*_ST_.

*F*_ST_ is a relative measure of population differentiation (Cruickshank & Hahn, 2014) and was outlined along with other *F*‐statistics by Sewall Wright (Wright, 1949). Estimates of *F*_ST_ have been used for many novel applications, such as examining parallel adaptation in sticklebacks (Hohenlohe, Bassham, Etter, & Cresko, 2010), introgression in canaries (Lopes et al., 2016) and local adaptation in high‐altitude populations of Tibetans (Peng et al., 2011; Xu et al., 2011). Recent work has used estimates of *F*_ST_ to identify and describe the divergence between relatively homomorphic sex chromosomes (Bergero, Gardner, Bader, Yong, & Charlesworth, 2019; Böhne et al., 2019; Conte et al., 2017; Dixon et al., 2018; Fontaine et al., 2017; Gammerdinger et al., 2014, 2016, 2018, 2019; Kirkpatrick & Guerrero, 2014; Natri, Shikano, & Merilä, 2013; Rodrigues & Dufresnes, 2017; Toups et al., 2018; Veltsos et al., 2019). However, there is a discrepancy within the literature regarding the expected estimate of *F*_ST_ for a fixed difference between the X‐ and Y‐chromosome. When comparing males and females for an allele that is either fixed on the X‐ or Y‐chromosome of an XY pair, some studies expect an *F*_ST_ estimate of $0.33\bar{3}$ (Gammerdinger et al., 2014, 2016, 2018, 2019; Kirkpatrick & Guerrero, 2014; Toups et al., 2018), while other studies expect an *F*_ST_ estimate of 0.5 (Böhne et al., 2019; Fontaine et al., 2017; Rodrigues & Dufresnes, 2017). The difference in these expectations is not typically justified, nor is the specific estimator of *F*_ST_ employed stated, leading to some confusion in the field.

A recent study highlighted inconsistencies between different estimators of *F*_ST_ (Berner, 2019) and in particular pointed out that, for an SNP fixed on the Y‐chromosome, one common estimator of *F*_ST_ yields a value of $0.33\bar{3}$ (Nei, 1973) while another yields 0.5 (Weir & Cockerham, 1984). How other popular estimators behave for an SNP that is alternatively fixed between the X‐ and Y‐chromosome, and, importantly, why these discrepancies arise, have yet to be systematically reviewed.

Here, we aim to clarify why such discrepancies in the expected estimates of *F*_ST_ can arise when comparing males and females for alternatively fixed alleles between the X‐ and Y‐chromosome. Note that this difference in expectations of *F*_ST_ is symmetric for ZW systems, so this analysis will only describe an XY system. Importantly, while this analysis focuses on the specific case of sex chromosomes, we also illustrate that the difference between *F*_ST_ estimators can be substantial for a wide range of allele frequencies, making direct comparisons of *F*_ST_ estimates between studies problematic in many contexts. Last, we apply a variety of population genetics software packages, which often generically refer to *F*_ST_, to estimate *F*_ST_ for alternatively fixed alleles between the X‐ and Y‐chromosome under various sampling schemes. Because these programs use different estimators and corrections for sample size and composition, a diverse range of expected *F*_ST_ values can be recovered (0.16–0.67) and, as a result, further complicates the interpretation of experimental studies that use *F*_ST_ to assess sex chromosome differentiation.

## 2 METHODS

We evaluated estimators of *F*_ST_ across different commonly used software packages, including vcftools version 0.1.15 (Danecek et al., 2011), arlequin 3.5 (Excoffier & Lischer, 2010), genepop 1.0.5 (Rousset, 2008), popgenome 2.61 (Pfeifer, Wittelsbürger, Ramos‐Onsins, & Lercher, 2014), hierfstat 0.04‐29 (Goudet, 2005), diversity 1.9.90 (Keenan, McGinnity, Cross, Crozier, & Prodöhl, 2013) and dnasp 6.12.03 (Rozas et al., 2017). Programs that used R were run on R 3.5.1 (R Core Team, 2016). Scripts for software packages that did not have a GUI are provided in File S1. During our use of these software packages, we analysed the effect of sample sizes on *F*_ST_ estimators. To perform this analysis, we created mock VCF files containing 20 fixed differences between the X‐ and Y‐chromosome for males and females (File S1) following the defined VCF format (Danecek et al., 2011). When necessary, we used pgdspider (Lischer & Excoffier, 2012) to convert our mock VCF files into fasta, arlequin, fstat and genepop formats.

## 3 WRIGHT’S *F*_ST_

Since Wright introduced *F*_ST_ (Wright, 1949), it has been unclear if this definition represents a parameter or an estimate of the parameter (Hahn, 2018; Holsinger & Weir, 2009). Nonetheless, *F*_ST_ for a biallelic system is most traditionally described as:

(1) $$F_{\text{ST}}=\frac{\sigma_{p}^{2}}{\bar{p}\bar{q}}$$

where $\sigma_{p}^{2}$ is the variance in the allele frequency for *p* and $\bar{p}$ and $\bar{q}$ are the average allele frequencies across the subpopulations for *p* and *q*, respectively (Hedrick, 2005; Weir & Cockerham, 1984). When comparing the sex chromosomes of males and females, we will define subpopulation 1 to be males and subpopulation 2 to be females, while *p* is the frequency of the allele on the X‐chromosome and *q* is the frequency of the allele on the Y‐chromosome (Table 1). When using Equation 1 and the values in Table 1, the resulting *F*_ST_ is $0.33\bar{3}$.

**TABLE 1:** Description of values for *p* and *q* in males and females

	Subpopulation 1 (males)	Subpopulation 2 (females)	Average of the subpopulations	*F*_ST_
*p* (frequency of the allele fixed on the X‐chromosome)	*p*_1_ = 0.5	*p_2_* = 1	$\bar{p}$ = 0.75	$0.33\bar{3}$
*q* (frequency of the allele fixed on the Y‐chromosome)	*q*_1_ = 0.5	*q_2_* = 0	$\bar{q}$ = 0.25	

## 4 ESTIMATORS OF *F*_ST_

In practice, the parameter values for allele frequencies are unknown and thus many methods have been proposed to estimate *F*_ST_. Here, we contrast two common estimators of *F*_ST_, which we will denote as ${\hat{F}}_{{\text{ST}}_{\text{Nei}}}$ and ${\hat{F}}_{{\text{ST}}_{\text{Hudson}}}$. The difference between these estimators has been previously discussed by others (Bhatia, Patterson, Sankararaman, & Price, 2013; Charlesworth, 1998), but not specifically in the context of sex chromosomes. While some estimators handle multiple alleles and multiple subpopulations, we will be considering only the biallelic state for two subpopulations. We make these simplifications because they provide a direct comparison between estimators and they reflect a situation with alternatively fixed alleles on the X‐ and Y‐chromosome when comparing males and females. Also, we will focus on the example of a fixed difference between the X‐ and Y‐chromosome because it is the fundamental component of these elevated estimates of *F*_ST_. However, *F*_ST_ estimates for various degrees of difference in the allele frequencies between males and females, going from equal frequencies in both sexes to alternatively fixed differences between the X‐ and Y‐chromosome, can be found in Figure S1. In an empirical study, the identification of an XY system would typically show a region overrepresented with these fixed differences between the X‐ and Y‐chromosome.

### 4.1 Nei's estimator of *F*_ST_

One estimator of *F*_ST_ comes from Nei (1973) and is often referred to as *G*_ST_. *G*_ST_ uses heterozygosity data to estimate *F*_ST_. *G*_ST_ quantifies the difference between the total heterozygosity of the population and the average heterozygosity of the subpopulations and normalizes this difference by the total heterozygosity of the population. It was defined by Nei (1973) as:

(2) $$G_{\text{ST}}=\frac{H_{T}‐H_{S}}{H_{T}}$$

When considering two alleles in two subpopulations, this estimator can be simplified to:

(3) $${\hat{F}}_{{\text{ST}}_{\text{Nei}}}=\frac{{\left(p_{1}‐p_{2}\right)}^{2}}{\left(p_{1}+p_{2}\right)\left(q_{1}+q_{2}\right)}$$

Thus, by comparing males and females for an allele that is alternatively fixed on the X‐ and Y‐chromosome using Nei’s (1973) estimator, the expected estimate of *F*_ST_ is $0.33\bar{3}$.

A similar estimator of *F*_ST_, called *γ*_ST_, is generally used in the context of haplotypes and estimates *F*_ST_ using nucleotide diversity. Nucleotide diversity, *π*, is the mean number of nucleotide differences between two randomly selected sequences from a population. *γ*_ST_ is described as the difference between the total nucleotide diversity of the population and the average nucleotide diversity of the subpopulations normalized to the total nucleotide diversity of the population (Nei, 1982). Nei (1982) defined *γ*_ST_ as:

(4) $$\gamma_{\text{ST}}=\frac{\pi_{T}‐\pi_{S}}{\pi_{T}}$$

where *π*_T_ the total nucleotide diversity of the population and *π*_S_ is the mean of the subpopulations’ nucleotide diversities. Notably, when considering a single, biallelic SNP, *G*_ST_ and *γ*_ST_ are equivalent (Nei, 1982). As a result, we will describe nucleotide diversities in terms of *p* and *q*, since nucleotide diversities and heterozygosities are equivalent for a SNP. We introduce *γ*_ST_ because it will lead to the most direct comparison between Nei’s (1973) estimator and Hudson, Slatkin, et al. (1992) estimator in the next section.

*π*_T_ estimates nucleotide diversity from $\bar{p}$ and $\bar{q}$, the mean of *p* and *q* across the subpopulations, respectively. Importantly, *π*_T_ makes comparisons between all alleles in the whole population. When considering a biallelic SNP in two subpopulations, *π*_T_ in Nei’s (1982) estimator can be simplified to:

(5) $$\pi_{T}=\frac{\left(p_{1}+p_{2}\right)\left(q_{1}+q_{2}\right)}{2}$$

Using the values from Table 1, we can estimate *π*_T_ as 0.375. The nucleotide diversity, *π*, of each subpopulation can be computed using Nei and Li’s (1979) definition for this statistic and averaged together to become *π*_S_. For a biallelic SNP in two subpopulations, *π*_S_ can be simplified to:

(6) $$\pi_{S}=p_{1}q_{1}+p_{2}q_{2}$$

When utilizing the values in Table 1, *π*_S_ is 0.25 and therefore Equation 4 recovers an expected estimate of *F*_ST_ to be $0.33\bar{3}$.

### 4.2 Hudson, Slatkin and Maddison's estimator of *F*_ST_

A second, alternative estimator of *F*_ST_ comes from Hudson, Slatkin, et al. (1992). This estimator considers the difference in the average nucleotide diversity between subpopulations and the average subpopulation nucleotide diversity and then normalizes this difference to the average nucleotide diversity between subpopulations. This estimator of *F*_ST_ is defined as:

(7) $${\hat{F}}_{{\text{ST}}_{\text{Hudson}}}=\frac{\pi_{B}‐\pi_{W}}{\pi_{B}}$$

where *π*_W_ is similar to Nei’s (1982) *π*_S_, except *π*_W_ excludes pairwise comparisons of haplotypes against themselves and is thus dependent on the subpopulation sample sizes. *π*_W_ can be expressed as:

(8) $$\pi_{W}=p_{1}q_{1}+p_{2}q_{2}+\frac{p_{1}q_{1}}{2n_{1}‐1}+\frac{p_{2}q_{2}}{2n_{2}‐1}$$

where *n*_1_ and *n*_2_ are the number of diploid individuals sampled from subpopulation 1 and 2, respectively. (A full derivation that can be found in the supplementary information of Bhatia et al. (2013). Note that in the Bhatia et al. (2013) derivation, *n*_1_ and *n*_2_ represent allele counts not diploid individual counts as are used here). As the subpopulation sample sizes go to infinity, *π*_W_ will approach *π*_S_ and thus the difference between *π*_S_ and *π*_W_ is often negligible with large subpopulation sample sizes. *π*_B_ is an alternative estimator of nucleotide diversity, defined by Nei and Li (1979) as *π_XY_*, and it can be quite numerically different from *π*_T_. *π_XY_* is the mean number of nucleotide differences between two randomly selected DNA sequences, each of which is drawn from separate subpopulations. In the case of a biallelic SNP in two subpopulations, Hudson, Slatkin, et al. (1992) estimator for *π*_B_ can be rewritten as:

(9) $$\pi_{B}=p_{1}q_{2}+p_{2}q_{1}$$

The values in Table 1 yield an estimate of *π*_B_ to be 0.5. As subpopulation sample sizes approach infinity, estimating *F*_ST_ for a biallelic locus in two subpopulations with this estimator can be written as:

(10) $${\hat{F}}_{{\text{ST}}_{\text{Hudson}}}=\frac{{\left(p_{1}‐p_{2}\right)}^{2}}{p_{1}q_{2}+p_{2}q_{1}}$$

**FIGURE 1:**
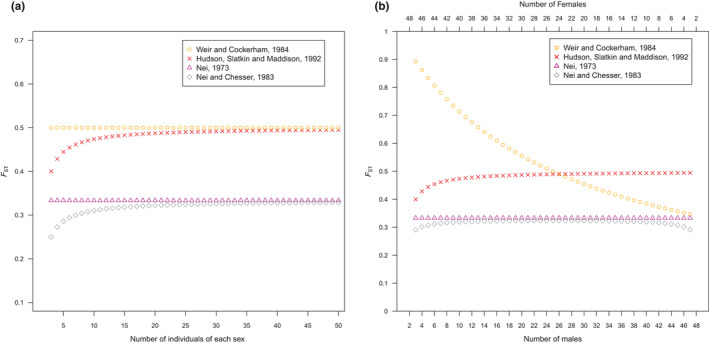
Various estimates of *F*_ST_ for a fixed difference between the X‐ and Y‐chromosome when (a) using equal subpopulation sample sizes for two subpopulations, males and females, and (b) using unequal subpopulation sample sizes for the two subpopulations, males and females, while keeping the total sample size constant

As subpopulation sizes go to infinity and using either Equation 7 or 10 with the values in Table 1, we arrive at an estimate of *F*_ST_ approaching 0.5. *F*_ST_ estimates for finite sample sizes using this estimator are demonstrated in Figure 1. Importantly, regardless of the subpopulation sample sizes employed, Nei’s (1973) estimator and Hudson, Slatkin, et al. (1992) estimator are always quite different for the case of sex chromosomes (Figure 1).

### 4.3 Why is there a difference in the expected estimate of F_ST_?

**FIGURE 2:**
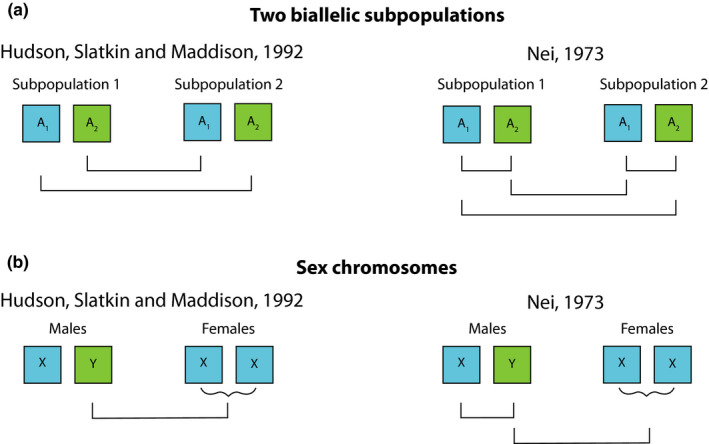
Comparison of the nonzero components of *π*_B_ in Hudson, Slatkin, et al. (1992) estimator and *π*_T_ in Nei’s (1973) estimator for biallelic SNPs in (a) two subpopulations and (b) an XY system. Each bar under the alleles represents a nonzero comparison that occurs in the formulation of *π*_B_ or *π*_T_. The curly bracket beneath females in the sex chromosome comparison illustrates that females are homomorphic for this allele despite being diploid and thus only one nonzero comparison is made

**FIGURE 3:**
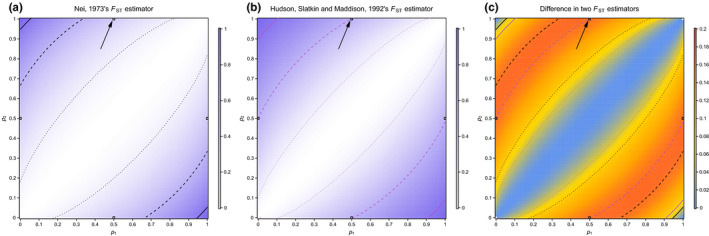
Visualizations of Nei (1973), Hudson, Slatkin, et al. (1992) and the difference between the two estimators. (a) Estimates of *F*_ST_ using the Nei (1973) estimator with white being no differentiation and dark blue being complete differentiation. (b) Estimates of *F*_ST_ using the Hudson, Slatkin, et al. (1992) estimator given infinitely large subpopulation sizes with white being no differentiation and dark blue being complete differentiation. (c) A heatmap of the difference between Hudson, Slatkin, et al. (1992) estimator and Nei’s (1973) estimator for *F*_ST_ (Hudson, Slatkin, et al. (1992) minus Nei (1973)) given infinitely large subpopulation sample sizes and the allele frequencies of *p* in subpopulations 1 and 2. Warmer colours show more difference between the estimators, while cooler colours show less difference between the estimators. Because the assignment of *p* and *q* along with subpopulation 1 and 2 is arbitrary, we have placed black boxes at all of the locations that could fit the description of a fixed difference between the X‐ and Y‐chromosome and provided an arrow to the scenario we outlined in Table 1. Dotted lines show an *F*_ST_ estimate equal to 0.1, dashed lines show an *F*_ST_ estimate equal to 0.5 and solid lines show an *F*_ST_ estimate equal to 0.9. Black dotted, dashed and solid lines are used to signify Nei’s (1973) estimator in panels (a) and (c), while purple dotted, dashed and solid lines are used to signify Hudson, Slatkin, et al. (1992) estimator in panels (b) and (c)

By comparing Equations 4 and 7 with large subpopulation sample sizes, it is clear that the important difference in Nei’s (1973, 1982) estimator and Hudson, Slatkin, et al. (1992) estimator arises in how they handle *π*_T_ and *π*_B_. Nei’s (1982) *π*_T_ uses two randomly drawn sequences from the population as a whole, while Hudson, Slatkin, et al. (1992) *π*_B_ requires that the two randomly drawn sequences be from the separate subpopulations. Figure 2 illustrates the difference between these two estimators for (a) a biallelic SNP present on an autosome in two subpopulations and for (b) sex chromosomes when comparing males and females. Figure 3 shows the *F*_ST_ estimates produced from Nei’s (1973) estimator and Hudson, Slatkin, et al. (1992) estimator when considering infinitely large subpopulation sample sizes, as well as the difference between these two estimators. Interestingly, the regions corresponding to an SNP that is alternatively fixed between the X‐ and Y‐chromosome are among the regions where the difference in these estimators is highest. However, these estimators can differ substantially across the range of plausible allele frequencies observed in two subpopulations. For example, SNPs that are not yet fixed differences between the X‐ and Y‐chromosome also show this discordance between *F*_ST_ estimators (Figure 3; Figure S1). Additionally, the difference in these two *F*_ST_ estimates when *p*_1_ is 0.20, *p*_2_ is 0.80 and both subpopulation samples sizes are 20 is slightly larger than the difference produced by sex chromosomes. This importantly illustrates that the disagreement in *F*_ST_ estimators is not the byproduct of the unique scenario of sex chromosomes, but is a disagreement that many researchers using *F*_ST_ estimators should consider.

### 4.4 Additional corrections to *F*_ST_

There are several estimators of *F*_ST_ that attempt to provide corrections for sampling biases and can cause further deviations from the expected estimate of *F*_ST_. Some estimators, such as that proposed by Hudson, Slatkin, et al. (1992), change with the total number of individuals sampled (Hudson, Boos, & Kaplan, 1992; Hudson, Slatkin, et al., 1992; Nei & Chesser, 1983) (Figure 1a; Table 2). Additionally, some estimators change as the proportion of individuals sampled from each subpopulation changes even when the total number of individuals sampled is held constant (Hudson, Boos, et al., 1992; Hudson, Slatkin, et al., 1992; Nei & Chesser, 1983; Weir & Cockerham, 1984) (Figure 1b; Table 2).

**TABLE 2:** Software packages for estimating *F*_ST_ and their estimates using mock input. These input files contained fixed differences between the X‐ and Y‐chromosome for various sample sizes of males and females

Package (version)	Option	5 Males and 5 females	10 Males and 10 females	20 Males and 20 females	5 Males and 15 females	15 Males and 5 females	Referenced estimator
vcftools (0.1.15)	weir‐fst‐pop	0.5	0.5	0.5	0.667	0.4	Weir and Cockerham, (1984)
popgenome (2.61)	F_ST.stats: nucleotide.F_ST	0.444	0.474	0.487	0.444	0.483	Hudson, Slatkin, et al. (1992)
	F_ST.stats: nuc.F_ST.pairwise	0.444	0.474	0.487	0.444	0.483	Hudson, Slatkin, et al. (1992)
	F_ST.stats: Nei.G_ST	0.333	0.333	0.333	0.333	0.333	Nei (1973)
	F_ST.stats: Nei.G_ST.pairwise	0.333	0.333	0.333	0.333	0.333	Nei (1973)
	F_ST.stats: Hudson.H_ST	0.296	0.316	0.325	0.45	0.163	Hudson, Boos, et al. (1992)^a^
	F_ST.stats: Hudson.G_ST	0.286	0.310	0.322	0.378	0.195	Hudson, Boos, et al. (1992)^b^ ^,^ ^c^
diversity (1.9.90)	diffCalc(fst = TRUE)	0.5	0.5	0.5	0.667	0.4	Weir and Cockerham (1984)
	diffCalc()	0.286	0.310	0.322	0.3023	0.3023	Nei and Chesser (1983)
hierfstat (0.04–29)	pairwise.fst	0.333	0.333	0.333	0.429	0.2	Nei (1973)^c^
	genet.dist(method = Nei87)	0.5	0.5	0.5	0.5	0.5	Nei (1987)^d^
	pairwise.neifst	0.5	0.5	0.5	0.5	0.5	Nei (1987)^d^
	basic.stats(fst)	0.333	0.333	0.333	0.333	0.333	Nei (1987)
	genet.dist(method = WC84)	0.5	0.5	0.5	0.667	0.4	Weir and Cockerham, (1984)
	pairwise.WCfst	0.5	0.5	0.5	0.667	0.4	Weir and Cockerham, (1984)
genepop (1.0.5)	Fst	0.5	0.5	0.5	0.667	0.4	Weir and Cockerham, (1984)
arlequin (3.5)	Compute pairwise FST	0.444	0.474	0.487	0.647	0.362	Excoffier, Smouse, and Quattro (1992)
dnasp (6.12.03)	Gene Flow and Genetic Differentiation: GST	0.286	0.310	0.322	0.378	0.194	Nei (1973)^b^ ^,^ ^c^
	Gene Flow and Genetic Differentiation: GammaSt	0.333	0.333	0.333	0.429	0.2	Nei (1982)^c^
	Gene Flow and Genetic Differentiation: Fst	0.444	0.474	0.487	0.444	0.483	Hudson, Slatkin, et al. (1992)

^a^ This implementation appears to use a $w_{i}\;=\;\frac{n_{i}\;‐\;2}{n_{1}\;+\;n_{2}\;‐\;4}$ weighting factor.

^b^ These estimates are most consistent with Nei and Chesser (1983), which is also discussed in Hudson, Boos, et al. (1992).

^c^ These metrics appear to use a $w_{i}\;=\;\frac{n_{i}}{n_{1}\;+\;n_{2}}$ weighting factor, while Nei (1982) and Nei and Chesser (1983) state that in most practices the subpopulations can be assumed to be weighted equally.

^d^ The referenced estimator is consistent with *F*′_ST_ in Nei (1987).

As similarly pointed out by Berner (2019), Weir and Cockerham’s (1984) estimator appears to respond dramatically to unequal numbers of males and females. As the proportion of the males in a constant sample size increases, the total variance of the sample increases and thus decreases the estimate of *F*_ST_ (Figure 1b). Regardless, in the case of a fixed difference between the X‐ and Y‐chromosome, $\bar{p}$ is defined as 0.75. Thus, there is little need to correct for subpopulation sample sizes because this differentiation is similar whether a single male and female are analysed or a very large number of each sex are considered. However, this type of sample size correction may be applicable when considering SNPs that are more frequent on the Y‐chromosome but not yet fixed or if it is unknown whether an SNP is alternatively fixed between the X‐ and Y‐chromosome.

An additional correction considers the number of subpopulations sampled (Hedrick, 2005; Weir & Cockerham, 1984). This correction is related to an infinite island model that assumes that the researcher is sampling a few subpopulations from a larger metapopulation. Because there are only two subpopulations, males and females, these corrections are probably unsuitable in this context.

Additionally, it has also been pointed out that *F*_ST_ underestimates differentiation at highly polymorphic loci, such as microsatellites (Charlesworth, 1998; Hedrick, 2005; Meirmans & Hedrick, 2011). Some estimators are particularly concerned with correcting for this bias (Hedrick, 2005; Meirmans & Hedrick, 2011); however, this correction for highly polymorphic loci is unlikely to be necessary for the biallelic locus in question.

While some of these corrections are probably inappropriate, authors may be using them as some software packages refer to their implementation generically as *F*_ST_ (Table 2). Table 2 and Figure 1 highlight the wide range of results that researchers could get for estimating *F*_ST_ depending on the subpopulation sample sizes and estimator employed. One may argue that any large deviation away from zero in *F*_ST_ estimates is sufficient enough evidence for sex chromosomes. However, estimates of *F*_ST_ that include the various aforementioned corrections may never reach $0.33\bar{3}$ or 0.5 (Table 2) and thus the expected maximum estimate of *F*_ST_ for a particular data set should ideally be considered and stated. Otherwise, deviations from the theoretical maximum due to these corrections could lead to an erroneous interpretation that there are no fixed differences between the X‐ and Y‐chromosome.

While the particular case of variants on sex chromosomes leads to some of the largest differences between these estimators, substantial differences can occur under alternative scenarios as well, and it would often be helpful to know how much of the variance between studies is driven by how *F*_ST_ is estimated. For instance, whether sexually antagonistic selection can explain the range of *F*_ST_ values that are found between males and females of different species (Cheng & Kirkpatrick, 2016; Flanagan & Jones, 2017; Lucotte, Laurent, Heyer, Ségurel, & Toupance, 2016; Wright et al., 2018; Wright, Rogers, Fumagalli, Cooney, & Mank, 2019) has recently been the subject of debate (Kasimatis, Nelson, & Phillips, 2017; Kasimatis, Ralph, & Phillips, 2019). While Kasimatis et al. (2019) compare Wright's *F*_ST_ to Weir and Cockerham's estimator of *F*_ST_, the variability introduced by the various estimators used in the previously cited experimental work (Hudson, Slatkin, et al., 1992; Nei, 1986; Weir, 1996; Wright, 1949) was not considered. In the future, we strongly urge researchers to justify their estimator, so that appropriate *F*_ST_ estimators are employed and estimates from various studies can be comparable.

## 5 CONCLUSIONS

When considering fixed differences between the X‐ and Y‐chromosome, we conclude that it is appropriate to use Nei’s (1973) estimator since it is most consistent with the work of Wright and others. However, both Nei’s (1973) and Hudson, Slatkin, et al. (1992) estimators are useful estimators of differentiation and there could be questions, such as those regarding polymorphisms that are not fully linked to the X‐ or Y‐chromosome, which are better answered with different estimators that implement some of the previously mentioned corrections. Moving forward, we encourage researchers to state which estimator they choose, their rationale for that choice and what the expected estimate of *F*_ST_ is for the data set they are investigating.

## AUTHOR CONTRIBUTIONS

W.J.G. conceived the commentary, drafted the manuscript and created the figures. M.A.T. and B.V. aided in drafting the manuscript and contributed intellectually to the commentary. M.A.T. also ran the software packages for the estimates of *F*_ST_ in Table 2.

## CONFLICTS OF INTEREST

The authors declare no conflicts of interest.

## Supporting information

Fig S1

## Data Availability

All data needed for reproducing these conclusions are within this work and Supporting Information.

## References

[men13210-bib-0001] Abbott, J. K. , Nordén, A. K. , & Hansson, B. (2017). Sex chromosome evolution: Historical insights and future perspectives. Proceedings of the Royal Society B: Biological Sciences, 284, 20162806 10.1098/rspb.2016.2806 PMC5443938 28469017

[men13210-bib-0002] Bachtrog, D. (2013). Y‐chromosome evolution: Emerging insights into processes of Y‐chromosome degeneration. Nature Reviews Genetics, 14, 113–124. 10.1038/nrg3366 PMC4120474 23329112

[men13210-bib-0003] Bachtrog, D. , Mank, J. E. , Peichel, C. L. , Kirkpatrick, M. , Otto, S. P. , Ashman, T.‐L. … Vamosi, J. C. (2014). Sex determination: Why so many ways of doing it? PLoS Biology, 12, e1001899 10.1371/journal.pbio.1001899 24983465 PMC4077654

[men13210-bib-0004] Bellott, D. W. , Hughes, J. F. , Skaletsky, H. , Brown, L. G. , Pyntikova, T. , Cho, T.‐J. … Page, D. C. (2014). Mammalian Y chromosomes retain widely expressed dosage‐sensitive regulators. Nature, 508, 494–499. 10.1038/nature13206 24759411 PMC4139287

[men13210-bib-0005] Bergero, R. , Gardner, J. , Bader, B. , Yong, L. , & Charlesworth, D. (2019). Exaggerated heterochiasmy in a fish with sex‐linked male coloration polymorphisms. Proceedings of the National Academy of Sciences of the United States of America, 116, 6924–6931. 10.1073/pnas.1818486116 30894479 PMC6452659

[men13210-bib-0006] Berner, D. (2019). Allele Frequency Difference *AFD* – An intuitive alternative to *F* _ST_ for quantifying population differentiation. Genes, 10, 308. 10.3390/genes10040308 PMC6523497 31003563

[men13210-bib-0007] Bhatia, G. , Patterson, N. , Sankararaman, S. , & Price, A. L. (2013). Estimating and interpreting *F* _ST_: The impact of rare variants. Genome Research, 23(9), 1514–1521. 10.1101/gr.154831.113 23861382 PMC3759727

[men13210-bib-0008] Blaser, O. , Grossen, C. , Neuenschwander, S. , & Perrin, N. (2012). Sex‐chromsome turnovers induced by deleterious mutation load. Evolution, 67, 635–645. 23461315 10.1111/j.1558-5646.2012.01810.x

[men13210-bib-0009] Böhne, A. , Weber, A. A.‐T. , Rajkov, J. , Rechsteiner, M. , Riss, A. , Egger, B. , & Salzburger, W. (2019). Repeated evolution versus common ancestry: Sex chromosome evolution in the Haplochromine Cichlid Pseudocrenilabrus philander. Genome Biology and Evolution, 11(2), 439–458. 10.1093/gbe/evz003 30649313 PMC6375353

[men13210-bib-0010] Charlesworth, B. (1998). Measures of divergence between populations and the effect of forces that reduce variability. Molecular Biology and Evolution, 15, 538–543. 10.1093/oxfordjournals.molbev.a025953 9580982

[men13210-bib-0011] Charlesworth, D. , Charlesworth, B. , & Marais, G. (2005). Steps in the evolution of heteromorphic sex chromosomes. Heredity, 95, 118–128. 10.1038/sj.hdy.6800697 15931241

[men13210-bib-0012] Cheng, C. , & Kirkpatrick, M. (2016). Sex‐specific selection and sex‐biased gene expression in humans and flies. PLoS Genetics, 12, 1–18. 10.1371/journal.pgen.1006170 PMC5033347 27658217

[men13210-bib-0013] Conte, M. A. , Gammerdinger, W. J. , Bartie, K. L. , Penman, D. J. , & Kocher, T. D. (2017). A high quality assembly of the Nile tilapia (*Oreochromis niloticus*) genome reveals the structure of two sex determination regions. BMC Genomics, 18, 341 10.1186/s12864-017-3723-5 28464822 PMC5414186

[men13210-bib-0014] Cortez, D. , Marin, R. , Toledo‐Flores, D. , Froidevaux, L. , Liechti, A. , Waters, P. D. , … Kaessmann, H. (2014). Origins and functional evolution of Y chromosomes across mammals. Nature, 508, 488–493. 10.1038/nature13151 24759410

[men13210-bib-0015] Cruickshank, T. E. , & Hahn, M. W. (2014). Reanalysis suggests that genomic islands of speciation are due to reduced diversity, not reduced gene flow. Molecular Ecology, 23, 3133–3157. 10.1111/mec.12796 24845075

[men13210-bib-0016] Danecek, P. , Auton, A. , Abecasis, G. , Albers, C. A. , Banks, E. , DePristo, M. A. , … Durbin, R. (2011). The variant call format and VCFtools. Bioinformatics, 27, 2156–2158. 10.1093/bioinformatics/btr330 21653522 PMC3137218

[men13210-bib-0017] Dixon, G. , Kitano, J. , & Kirkpatrick, M. (2018). The origin of a new sex chromosome by introgression between two stickleback fishes. Molecular Biology and Evolution, 36, 28–38. 10.1093/molbev/msy181 PMC6340465 30272243

[men13210-bib-0018] Excoffier, L. , & Lischer, H. E. L. (2010). Arlequin suite ver 3.5: A new series of programs to perform population genetics analyses under Linux and Windows. Molecular Ecology Resources, 10, 564–567. 10.1111/j.1755-0998.2010.02847.x 21565059

[men13210-bib-0019] Excoffier, L. , Smouse, P. E. , & Quattro, J. M. (1992). Analysis of molecular variance inferred from metric distances among DNA haplotypes: Application to Human mitochondrial DNA restriction data. Genetics, 131, 479–491. 1644282 10.1093/genetics/131.2.479 PMC1205020

[men13210-bib-0020] Flanagan, S. P. , & Jones, A. G. (2017). Genome‐wide selection components analysis in a fish with male pregnancy. Evolution, 71, 1096–1105. 10.1111/evo.13173 28067418

[men13210-bib-0021] Fontaine, A. , Filipovi, I. , Fansiri, T. , Hoffmann, A. A. , Cheng, C. , Kirkpatrick, M. … Lambrechts, L. (2017). Extensive genetic differentiation between homomorphic sex chromosomes in the mosquito vector, *Aedes aegypti* . Genome Biology and Evolution, 9, 2322–2335. 10.1093/gbe/evx171 28945882 PMC5737474

[men13210-bib-0022] Fraïsse, C. , Picard, M. A. L. , & Vicoso, B. (2017). The deep conservation of the Lepidoptera Z chromosome suggests a non‐canonical origin of the W. Nature Communications, 8, 1486 10.1038/s41467-017-01663-5 PMC5684275 29133797

[men13210-bib-0023] Gammerdinger, W. J. , Conte, M. A. , Acquah, E. A. , Roberts, R. B. , & Kocher, T. D. (2014). Structure and decay of a proto‐Y region in tilapia, *Oreochromis niloticus* . BMC Genomics, 15, 975 10.1186/1471-2164-15-975 25404257 PMC4251933

[men13210-bib-0024] Gammerdinger, W. J. , Conte, M. A. , Baroiller, J.‐F. , D’Cotta, H. , & Kocher, T. D. (2016). Comparative analysis of a sex chromosome from the blackchin tilapia, *Sarotherodon melanotheron* . BMC Genomics, 17, 808 10.1186/s12864-016-3163-7 27756226 PMC5070092

[men13210-bib-0025] Gammerdinger, W. J. , Conte, M. A. , Sandkam, B. A. , Penman, D. J. , & Kocher, T. D. (2019). Characterization of sex chromosomes in three deeply diverged species of Pseudocrenilabrinae (Teleostei: Cichlidae). Hydrobiologia, 832, 397–408. 10.1007/s10750-018-3778-6 PMC9162429 35665074

[men13210-bib-0026] Gammerdinger, W. J. , Conte, M. A. , Sandkam, B. A. , & Ziegelbecker, A. (2018). Novel sex chromosomes in three cichlid fishes from Lake Tanganyika. Journal of Heredity, 1, 12. 10.1093/jhered/esy003 29444291

[men13210-bib-0027] Goudet, J. (2005). HIERFSTAT, a package for R to compute and test hierarchical F‐statistics. Molecular Ecology Notes, 5, 184–186. 10.1111/j.1471-8286.2004.00828.x

[men13210-bib-0028] Hahn, M. W. (2018). Molecular population genetics. New York: Oxford University Press.

[men13210-bib-0029] Hedrick, P. W. (2005). A standardized genetic differentiation measure. Evolution, 59, 1633–1638. 10.1111/j.0014-3820.2005.tb01814.x 16329237

[men13210-bib-0030] Hohenlohe, P. A. , Bassham, S. , Etter, P. D. , & Cresko, W. A. (2010). Population genomics of parallel adaptation in threespine stickleback using sequenced RAD tags. PLoS Genetics, 6, e1000862 10.1371/journal.pgen.1000862 20195501 PMC2829049

[men13210-bib-0031] Holsinger, K. E. , & Weir, B. S. (2009). Genetics in geographically structured populations: Defining, estimating and interpreting F_ST_ . Nature Reviews Genetics, 10, 639–650. 10.1038/nrg2611 PMC4687486 19687804

[men13210-bib-0032] Hudson, R. R. , Boos, D. D. , & Kaplan, N. L. (1992). A statistical test for detecting geographic subdivision. Molecular Biology and Evolution, 9, 138–151. 1552836 10.1093/oxfordjournals.molbev.a040703

[men13210-bib-0033] Hudson, R. R. , Slatkin, M. , & Maddison, W. P. (1992). Estimation of levels of gene flow from DNA sequence data. Genetics, 589, 583–589. 10.1093/genetics/132.2.583 PMC1205159 1427045

[men13210-bib-0034] Huylmans, A. K. , Toups, M. A. , Macon, A. , Gammerdinger, W. J. , & Vicoso, B. (2019). Sex–biased gene expression and dosage compensation on the *Artemia franciscana* Z‐chromosome. Genome Biology and Evolution, 11:1033–1044. 30865260 10.1093/gbe/evz053 PMC6456005

[men13210-bib-0035] Kamiya, T. , Kai, W. , Tasumi, S. , Oka, A. , Matsunaga, T. , Mizuno, N. … Kikuchi, K. (2012). A trans‐species missense SNP in Amhr2 is associated with sex determination in the tiger pufferfish, *Takifugu rubripes* (fugu). PLoS Genetics, 8, e1002798 10.1371/journal.pgen.1002798 22807687 PMC3395601

[men13210-bib-0036] Kasimatis, K. R. , Nelson, T. C. , & Phillips, P. C. (2017). Genomic signatures of sexual conflict. Journal of Heredity, 108, 780–790. 10.1093/jhered/esx080 29036624 PMC5892400

[men13210-bib-0037] Kasimatis, K. R. , Ralph, P. L. , & Phillips, P. C. (2019). Limits to genomic divergence under sexually antagonistic selection. G3 Genes, Genomes, Genetics, 9(11), 3813–3824. 10.1534/g3.119.400711 31530636 PMC6829153

[men13210-bib-0038] Keenan, K. , McGinnity, P. , Cross, T. F. , Crozier, W. W. , & Prodöhl, P. A. (2013). diveRsity: An R package for the estimation and exploration of population genetics parameters and their associated errors. Methods in Ecology and Evolution, 4, 782–788.

[men13210-bib-0039] Kirkpatrick, M. , & Guerrero, R. (2014). Signatures of sex‐antagonistic selection on recombining sex chromosomes. Genetics, 197, 531–541. 10.1534/genetics.113.156026 24578352 PMC4063913

[men13210-bib-0040] Lischer, H. E. L. , & Excoffier, L. (2012). PGDSpider: An automated data conversion tool for connecting population genetics and genomics programs. Bioinformatics, 28, 298–299. 10.1093/bioinformatics/btr642 22110245

[men13210-bib-0041] Lopes, R. J. , Johnson, J. D. , Toomey, M. B. , Ferreira, M. S. , Araujo, P. M. , Melo‐Ferreira, J. … Carneiro, M. (2016). Genetic basis for red coloration in birds. Current Biology, 26, 1427–1434. 10.1016/j.cub.2016.03.076 27212400 PMC5125026

[men13210-bib-0042] Lucotte, E. A. , Laurent, R. , Heyer, E. , Ségurel, L. , & Toupance, B. (2016). Detection of allelic frequency differences between the sexes in humans: A signature of sexually antagonistic selection. Genome Biology and Evolution, 8, 1489–1500. 10.1093/gbe/evw090 27189992 PMC4898804

[men13210-bib-0043] Meirmans, P. G. , & Hedrick, P. W. (2011). Assessing population structure: F_ST_ and related measures. Molecular Ecology Resources, 11, 5–18. 10.1111/j.1755-0998.2010.02927.x 21429096

[men13210-bib-0044] Natri, H. M. , Shikano, T. , & Merilä, J. (2013). Progressive recombination suppression and differentiation in recently evolved neo‐sex chromosomes. Molecular Biology and Evolution, 30, 1131–1144. 10.1093/molbev/mst035 23436913 PMC3670740

[men13210-bib-0045] Nei, M. (1973). Analysis of gene diversity in subdivided populations. Proceedings of the National Academy of Sciences of the United States of America, 70, 3321–3323. 10.1073/pnas.70.12.3321 4519626 PMC427228

[men13210-bib-0046] Nei, M. (1982). Evolution of Human races at the gene level In Alan L. R. (Ed.), Human genetics, part A: The unfolding genome (pp. 167–181). New York: Liss. 7163193

[men13210-bib-0047] Nei, M. (1986). Definition and estimation of fixation indices. Evolution, 40, 643–645. 10.1111/j.1558-5646.1986.tb00516.x 28556335

[men13210-bib-0048] Nei, M. (1987). Molecular evolutionary genetics. New York: Columbia University Press.

[men13210-bib-0049] Nei, M. , & Chesser, R. K. (1983). Estimation of fixation indices and gene diversities. Annals of Human Genetics, 47, 253–259. 10.1111/j.1469-1809.1983.tb00993.x 6614868

[men13210-bib-0050] Nei, M. , & Li, W.‐H. (1979). Mathematical model for studying genetic variation in terms of restriction endonucleases. Proceedings of the National Academy of Sciences of the United States of America, 76, 5269–5273. 10.1073/pnas.76.10.5269 291943 PMC413122

[men13210-bib-0051] Pal, A. , & Vicoso, B. (2015). The X chromosome of Hemipteran insects: Conservation, dosage compensation and sex‐biased expression. Genome Biology and Evolution, 7, 3259–3268. 10.1093/gbe/evv215 26556591 PMC4700948

[men13210-bib-0052] Peng, Y. , Yang, Z. , Zhang, H. , Cui, C. , Qi, X. , Luo, X. … Su, B. (2011). Genetic variations in Tibetan populations and high‐altitude adaptation at the Himalayas. Molecular Biology and Evolution, 28, 1075–1081. 10.1093/molbev/msq290 21030426

[men13210-bib-0053] Pfeifer, B. , Wittelsbürger, U. , Ramos‐Onsins, S. E. , & Lercher, M. J. (2014). PopGenome: An efficient swiss army knife for population genomic analyses in R. Molecular Biology and Evolution, 31, 1929–1936. 10.1093/molbev/msu136 24739305 PMC4069620

[men13210-bib-0054] R Core Team . (2016). A language and environment for statistical computing. Vienna: R Foundation for Statistical Computing.

[men13210-bib-0055] Rice, W. R. (1987). The accumulation of sexually antagonistic genes as a selective agent promoting the evolution of reduced recombination between primitive sex chromosomes. Evolution, 41, 911–914. 10.1111/j.1558-5646.1987.tb05864.x 28564364

[men13210-bib-0056] Rodrigues, N. , & Dufresnes, C. (2017). Using conventional *F* ‐statistics to study unconventional sex‐chromosome differentiation. PeerJ, 5, e3207. 28462023 10.7717/peerj.3207 PMC5410149

[men13210-bib-0057] Roesti, M. , Moser, D. , & Berner, D. (2013). Recombination in the threespine stickleback genome — patterns and consequences. Molecular Ecology, 22, 3014–3027. 10.1111/mec.12322 23601112

[men13210-bib-0058] Rousset, F. (2008). GENEPOP’007: A complete re‐implementation of the GENEPOP software for Windows and Linux. Molecular Ecology Resources, 8, 103–106. 10.1111/j.1471-8286.2007.01931.x 21585727

[men13210-bib-0059] Rozas, J. , Ferrer‐Mata, A. , Sánchez‐DelBarrio, J. C. , Guirao‐Rico, S. , Librado, P. , Ramos‐Onsins, S. E. , & Sánchez‐Gracia, A. (2017). DnaSP 6: DNA sequence polymorphism analysis of large data sets. Molecular Biology and Evolution, 34, 3299–3302. 10.1093/molbev/msx248 29029172

[men13210-bib-0060] Toups, M. A. , Rodrigues, N. , Perrin, N. , & Kirkpatrick, M. (2018). A reciprocal translocation radically reshapes sex‐linked inheritance in the common frog. Molecular Ecology, 10.1111/mec.14990. PMC7558804 30576024

[men13210-bib-0061] van Doorn, G. S. , & Kirkpatrick, M. (2010). Transitions between male and female heterogamety caused by sex‐antagonistic selection. Genetics, 186, 629–645. 10.1534/genetics.110.118596 20628036 PMC2954476

[men13210-bib-0062] Veltsos, P. , Ridout, K. E. , Toups, M. A. , González‐Martínez, S. C. , Muyle, A. , Emery, O. , … Pannell, J. R. (2019). Early sex‐chromosome evolution in the diploid dioecious plant *Mercurialis annua* . Genetics, 212, 815–835. 31113811 10.1534/genetics.119.302045 PMC6614902

[men13210-bib-0063] Vicoso, B. , & Bachtrog, D. (2011). Lack of global dosage compensation in *Schistosoma mansoni*; a female‐heterogametic parasite. Genome Biology and Evolution, 3, 230–235. 10.1093/gbe/evr010 21317157 PMC3068002

[men13210-bib-0064] Vicoso, B. , & Bachtrog, D. (2013). Reversal of an ancient sex chromosome to an autosome in *Drosophila* . Nature, 499, 332–335. 10.1038/nature12235 23792562 PMC4120283

[men13210-bib-0065] Vicoso, B. , & Bachtrog, D. (2015). Numerous transitions of sex chromosomes in Diptera. PLoS Biology, 13, e1002078 10.1371/journal.pbio.1002078 25879221 PMC4400102

[men13210-bib-0066] Vicoso, B. , Kaiser, V. B. , & Bachtrog, D. (2013). Sex‐biased gene expression at homomorphic sex chromosomes in emus and its implication for sex chromosome evolution. Proceedings of the National Academy of Sciences of the United States of America, 110, 6453–6458. 10.1073/pnas.1217027110 23547111 PMC3631621

[men13210-bib-0067] Weir, B. S. (1996). Genetic data analysis II. Sunderland, MA: Sinauer Associates Inc.

[men13210-bib-0068] Weir, B. , & Cockerham, C. C. (1984). Estimating F‐statistics for the analysis of population structure. Evolution, 38, 1358–1370. 28563791 10.1111/j.1558-5646.1984.tb05657.x

[men13210-bib-0069] Wright, A. E. , Fumagalli, M. , Cooney, C. R. , Bloch, N. I. , Vieira, F. G. , Buechel, S. D. … Mank, J. E. (2018). Male‐biased gene expression resolves sexual conflict through the evolution of sex‐specific genetic architecture. Evolution Letters, 2, 52–61. 10.1002/evl3.39 30283664 PMC6089503

[men13210-bib-0070] Wright, A. E. , Rogers, T. F. , Fumagalli, M. , Cooney, C. R. , & Mank, J. E. (2019). Phenotypic sexual dimorphism is associated with genomic signatures of resolved sexual conflict. Molecular Ecology, 28, 2860–2871. 10.1111/mec.15115 31038811 PMC6618015

[men13210-bib-0071] Wright, S. (1949). The genetical structure of populations. Annals of Eugenics, 15, 323–354. 10.1111/j.1469-1809.1949.tb02451.x 24540312

[men13210-bib-0072] Xu, S. , Li, S. , Yang, Y. , Tan, J. , Lou, H. , Jin, W. … Jin, L. (2011). A genome‐wide search for signals of high‐altitude adaptation in Tibetans. Molecular Biology and Evolution, 28, 1003–1011. 10.1093/molbev/msq277 20961960

