# Supplementary figures and images for: Disagreement in F ST estimators: A case study from sex chromosomes
Source: Mol Ecol Resour. 2020 Jul 6;20(6):1517–25. doi: 10.1111/1755-0998.13210 (PMC7689734; doi:10.1111/1755-0998.13210)

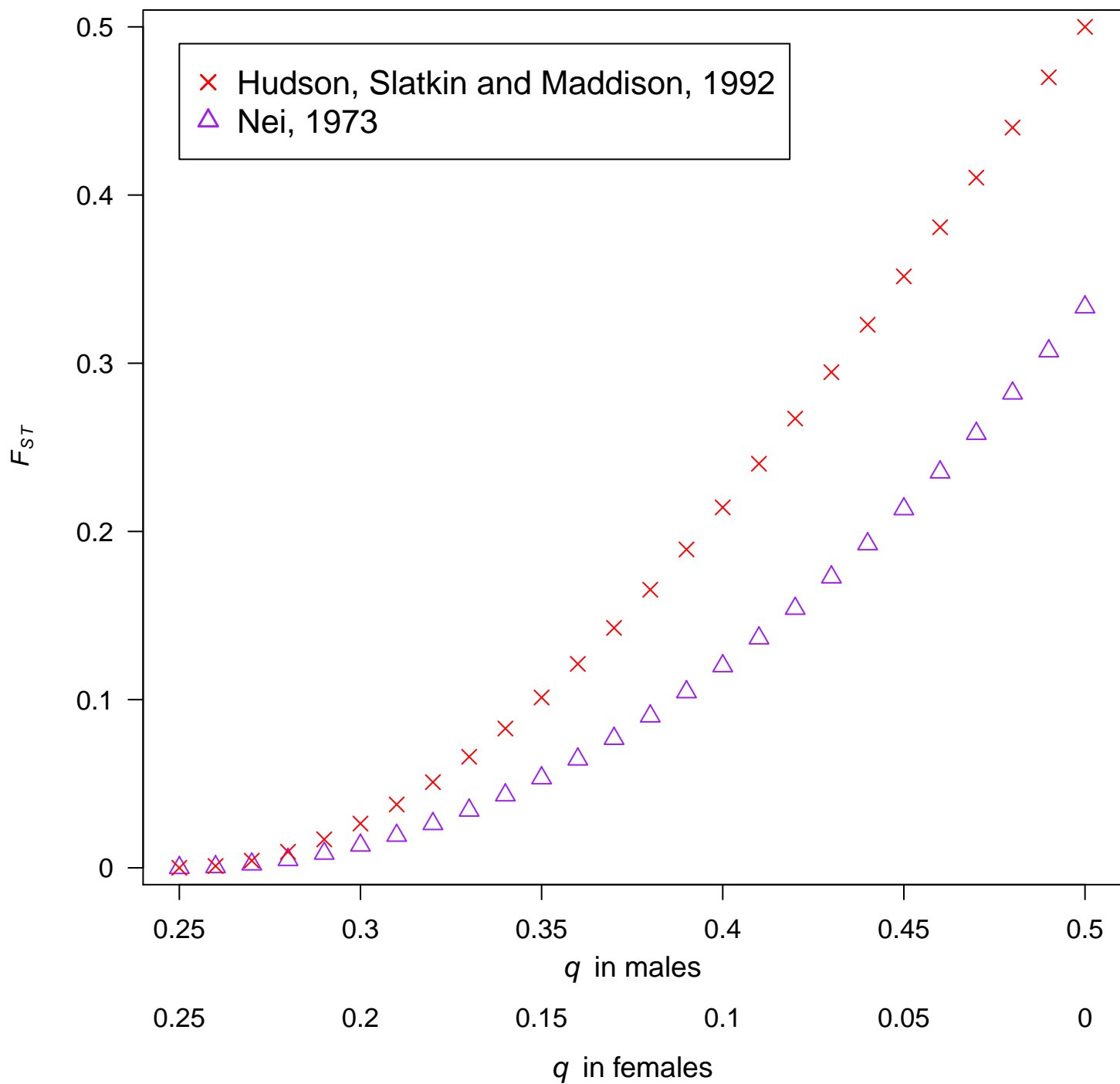

Supplement: Supplementary file 1 — Fig S1 [file MEN-20-1517-s001.pdf]
